# Can CHA_2_DS_2_-VASc and HAS–BLED Foresee the Presence of Cerebral Microbleeds, Lacunar and Non-Lacunar Infarcts in Elderly Patients With Atrial Fibrillation? Data From Strat–AF Study

**DOI:** 10.3389/fneur.2022.883786

**Published:** 2022-05-13

**Authors:** Elisa Bianconi, Giulia Del Freo, Emilia Salvadori, Carmen Barbato, Benedetta Formelli, Francesca Pescini, Giovanni Pracucci, Cristina Sarti, Francesca Cesari, Stefano Chiti, Stefano Diciotti, Anna Maria Gori, Chiara Marzi, Enrico Fainardi, Betti Giusti, Rossella Marcucci, Bruno Bertaccini, Anna Poggesi

**Affiliations:** ^1^Department of Statistics, Computer Science, Applications ≪ G. Parenti ≫, University of Florence, Florence, Italy; ^2^NEUROFARBA Department, Neuroscience Section, University of Florence, Florence, Italy; ^3^Don Carlo Gnocchi Foundation, Milan, Italy; ^4^Stroke Unit, Careggi University Hospital, Florence, Italy; ^5^Central Laboratory, Careggi University Hospital, Florence, Italy; ^6^Department Health Professions, U.O. Research and Development, Careggi University Hospital, Florence, Italy; ^7^Department of Electrical, Electronic, and Information Engineering “Guglielmo Marconi”, University of Bologna, Cesena, Italy; ^8^Department of Experimental and Clinical Medicine, University of Florence, Florence, Italy; ^9^Atherothrombotic Diseases Center, Careggi University Hospital, Florence, Italy; ^10^Institute of Applied Physics “Nello Carrara” (IFAC), National Research Council of Italy (CNR), Florence, Italy; ^11^Neuroradiology Unit, Department of Experimental and Clinical Biomedical Sciences, Careggi University Hospital, University of Florence, Florence, Italy

**Keywords:** atrial fibrillation, anticoagulation, stroke, intracerebral hemorrhage, cerebral small vessel disease, brain MRI, CHA_2_DS_2_-VASc scale, HAS-BLED scale

## Abstract

Anticoagulants reduce embolic risk in atrial fibrillation (AF), despite increasing hemorrhagic risk. In this context, validity of congestive heart failure, hypertension, age ≥ 75 years, diabetes, stroke, vascular disease, age 65–74 years and sex category (CHA_2_DS_2_-VASc) and hypertension, abnormal renal/liver function, stroke, bleeding history or predisposition, labile international normalized ratio, elderly, drugs/alcohol concomitantly (HAS–BLED) scales, used to respectively evaluate thrombotic and hemorrhagic risks, is incomplete. In patients with AF, brain MRI has led to the increased detection of “asymptomatic” brain changes, particularly those related to small vessel disease, which also represent the pathologic substrate of intracranial hemorrhage, and silent brain infarcts, which are considered risk factors for ischemic stroke. Routine brain MRI in asymptomatic patients with AF is not yet recommended. Our aim was to test predictive ability of risk stratification scales on the presence of cerebral microbleeds, lacunar, and non-lacunar infarcts in 170 elderly patients with AF on oral anticoagulants. *Ad hoc* developed R algorithms were used to evaluate CHA_2_DS_2_-VASc and HAS–BLED sensitivity and specificity on the prediction of cerebrovascular lesions: (1) Maintaining original items' weights; (2) augmenting weights' range; (3) adding cognitive, motor, and depressive scores. Accuracy was poor for each outcome considering both scales either in phase 1 or phase 2. Accuracy was never improved by the addition of cognitive scores. The addition of motor and depressive scores to CHA_2_DS_2_-VASc improved accuracy for non-lacunar infarcts (sensitivity = 0.70, specificity = 0.85), and sensitivity for lacunar–infarcts (sensitivity = 0.74, specificity = 0.61). Our results are a very first step toward the attempt to identify those elderly patients with AF who would benefit most from brain MRI in risk stratification.

## Introduction

Atrial fibrillation (AF) is the most common arrhythmia encountered in clinical practice, and its prevalence is strictly age-related as discussed in the following: ~1% of patients with AF are younger than 60 years of age, whereas up to 12% of patients with AF are 75–84 years of age, with even higher prevalence over 80 years old (1). Atrial fibrillation is associated with high morbidity and mortality, with an increased risk of stroke and thromboembolism. Thromboprophylaxis with oral anticoagulation is effective in reducing stroke risk in patients with AF and is currently recommended by guidelines for patients with moderate/high risk of stroke (1, 2).

To aid decision-making for thromboprophylaxis, several clinical risk stratification schemes have been developed. To estimate thromboembolic risk, guidelines mainly propose the use of congestive heart failure, hypertension, age ≥75 years, diabetes, stroke, vascular disease, age 65–74 years, and sex category (CHA_2_DS_2_-VASc) (3– 5). Benefits of anticoagulation have to be balanced against the risk of bleeding, with intracranial hemorrhage being the most feared one. Hypertension, abnormal renal/liver function, stroke, bleeding history or predisposition, labile international normalized ratio, elderly, and drugs/alcohol concomitantly (HAS-BLED) can be used to assess bleeding risk in patients for whom anti-coagulation is being considered (2, 6). Available stroke and bleeding risk stratification schemes are aimed at identifying patients who may benefit most from oral anticoagulation, and also might orient in the choice of the type of oral anticoagulant (7). Such schemes currently rely on just clinical information; the validity of which remains controversial and needs to be improved.

In patients with AF, neuroimaging, mainly MRI, has led to increased detection of “asymptomatic” brain changes, such as those related to small vessel disease (SVD), which also represent the pathologic substrate of intracerebral hemorrhage, and silent brain infarcts, which double the risk of ischemic stroke (8, 9). Such imaging findings are proposed as additional markers for risk stratification (10). Conclusive evidence about their predictive value in single patients can only come from adequately powered large prospective follow-up studies, and some efforts are already under way. In patients with previous stroke and atrial fibrillation, Clinical Relevance Of Microbleeds In Stroke (CROMIS-2) and Hemorrhage Predicted by Resonance in Patients Receiving Oral Anticoagulants (HERO) studies have demonstrated that the presence of severe SVD, particularly cerebral microbleeds (CMBs), is a strong predictor of intracerebral hemorrhage (11, 12). Despite this, debate is ongoing on how to consider such “brain” information in decision making for anticoagulants (13, 14), and considering the MRI-associated costs, the routine use of brain MRI in asymptomatic patients with AF is still not recommended.

In line with precision medicine, a cost-effective strategy would be the development of a method able to classify patients into subgroups that differ in their susceptibility to thromboembolic and bleeding risks, and then decide to collect “brain” information only on those who will benefit (15).

Our aim was to test the predictive ability of the currently recommended risk stratification scales, CHA_2_DS_2_-VASc and HAS–BLED, on the presence of brain MRI lesions related to SVD and non-lacunar infarcts in elderly patients with AF on oral anticoagulants.

## Materials and Methods

Stratification of cerebral bleeding risk in AF, http://www.strat-af.it (Strat-AF) is an observational, prospective, single-center hospital-based study enrolling elderly patients with AF, referred from the outpatient clinic Center of Thrombosis of Careggi University Hospital in Florence where they are followed for the management of oral anticoagulation therapy in primary or secondary prevention of thromboembolic events. Strat-AF study design and methodology have been previously described (16). In brief, the main aim of Strat-AF study is to evaluate the added value of circulating and brain MRI biomarkers on the prediction of cerebral bleeding risk. For this purpose, patients affected by AF aged ≥65 years on oral anticoagulants and no contraindication to MRI were enrolled. Ethical approval was obtained by our Ethics Committee, and all participants gave written informed consent.

Among collected clinical information, for the purpose of this paper, demographic characteristics (age, sex, and years of education), vascular risk factors and comorbidities (hypertension, diabetes, dyslipidemia, physical activity, smoking habits, alcohol consumption, stroke, peripheral arterial disease, ischemic heart disease, myocardial infarction, and heart failure) were used for the analyses.

Global cognitive efficiency was assessed by the Montreal Cognitive Assessment (MoCA) (17). Italian normative data were used to adjust MoCA raw scores for demographics, and to convert adjusted scores into an ordinal 5-point scale according to the equivalent score non-parametric norming method. Equivalent scores method is based on percentiles distributions, and scores range from ES = 0 (impaired performance) to ES = 4 (optimal performance) (18, 19).

Short physical performance Battery (SPPB) was used to evaluate motor performance with a total score ranging from 0 (not able to walk) to 12 (best motor performance) (20).

The 15-item version of Geriatric Depression Scale (GDS) was used to evaluate depressive symptoms. The total score ranges from 0 to 15 (with each point indicating more severe depressive symptoms) (21).

The CHA_2_DS_2_-VASc and HAS–BLED scores were calculated based on the clinical information. The CHA_2_DS_2_-VASc scores range from 0 to 9 and are based on the following clinical information: Congestive heart failure (score 1), hypertension or antihypertensive therapy (score 1), age ≥75 years (score 2), diabetes (score 1), stroke (score 2), vascular disease (score 1), age 65–74 years (score 1), and sex category (female, score 1) (3). The HAS-BLED scale is used to estimate the bleeding risk, with scores ranging from 0 to 9, which was calculated based on the following clinical information: Uncontrolled hypertension (systolic blood pressure >160 mm Hg, score 1), abnormal renal and/or hepatic function (score 1 or 2), stroke (score 1), bleeding history or predisposition (score 1), labile international normalized ratio (score 1), age (score 1), and drugs or excessive alcohol drinking (score 1 or 2) (6).

All participants underwent brain MRI on a 1.5 T (Ingenia, Philips Healthcare, Best, The Netherlands). The MRI protocol included the following sequences: Sagittal T1-weighted spin–echo, coronal T2-weighted Turbo spin–echo; axial fluid-attenuated inversion recovery (FLAIR); axial gradient–echo T2^*^ (FFE); axial diffusion weighted imaging (DWI); sagittal magnetization prepared rapid acquisition gradient–echo 3D T1-weighted (T1 TFE 3D) followed by MultiPlanar reconstruction (MPR) in axial, coronal, and sagittal planes. Further details are reported in the methodology study (16).

For the purposes of the present analyses, the following brain MRI lesions were visually rated by a trained and experienced rater:

- Non-lacunar infarcts were assessed in terms of presence and number on T1 and T2 FLAIR sequences as cortical–subcortical lesions in vascular territories.
- The CMBs were detected and counted on axial gradient–echo T2 sequences and classified according to microbleed anatomical rating scale (MARS) (22).
- Lacunes of presumed vascular origin were detected and counted on T1 and T2 FLAIR sequences according to STRIVE criteria (23).

### Statistical Analyses

The descriptive analyses (frequencies and percentages or means and standard deviations) were carried out to describe the total cohort in terms of demographics, vascular risk factors, comorbidities, and motor, cognitive, and neuroimaging characteristics.

For statistical purposes, the following variables were dichotomized as follows:

- MoCA performance “impaired” (ES = 0) *vs*. “normal” (ES ≥ 1) (18).
- Motor performance “impaired” (SPPB score ≤ 10) *vs*. “normal” (SPPB score >10) (24).
- Depressive symptoms “present” (GDS score > 5) *vs*. “absent” (GDS score ≤ 5) (25).
- Non-lacunar infarcts absent *vs*. ≥ 1.
- Cerebral microbleeds absent *vs*. ≥ 1.
- Lacunar infarcts absent *vs*. ≥1.

The imaging variables were the three study outcomes for which we evaluated the predictive ability, in terms of sensitivity and specificity, of CHA_2_DS_2_-VASc and HAS–BLED. The two scales were evaluated separately.

### Algorithms in the R Environment

Version 4.1.0 of R (foundation for statistical computing) was used for the development of dedicated algorithms to test the possible predictive value of the two clinical scales on the presence of the three neuroimaging markers, according to the following phases:

#### Phase 1: Predictive Ability of CHA_2_DS_2_-VASc and HAS–BLED

In this phase, the effectiveness of CHA_2_DS_2_-VASc and HAS–BLED scales was tested in relation to the following three study outcomes: Non-lacunar infarcts, CMBs, and lacunar infarcts. Weights associated to each scale item were maintained as originally proposed in literature (3, 6). For both scales, the algorithm returned the sensitivity and the specificity computed by varying all possible scores that each scale can assume.

#### Phase 2: Changing Weights of CHA_2_DS_2_-VASc and HAS–BLED Items

Analyses of Phase 1 were repeated modifying weights assigned to the individual items of each scale. We assumed that the weight could change from 0 (excluded item) to 4 (maximum weight), and only integer values were used. A matrix containing all possible combinations of weights was created. Subsequently, the algorithm attributed the various combinations of weights to the variables and calculated, for each combination, the predictive capability using the Youden index (J) (26). The algorithm identified the combinations “frontier,” i.e., combinations of weights with the maximum Youden index. Then, among all the possible scores that can be assumed by each “frontier” combination, the algorithm looked for the score with the maximum Youden index associated. Such score will be used in prediction as a “cutoff” point, that is the bisection point between a prediction of absence of the outcome with respect to its presence. In relation to the “frontier” combinations and the relative “cutoffs” the relative sensitivity and specificity was calculated. At the end of the computational process, among the “frontier” combinations, we selected the ones with maximum sensitivity. In conclusion, for each scale, the algorithm returned performance indexes (sensitivity and specificity) of the best combinations of weights obtained by maximizing the sensitivity for each neuroimaging outcome.

#### Phase 3: Adding New Items to CHA_2_DS_2_-VASc and HAS–BLED

In the third phase, we tested the effect of the addition of three clinical variables to the scales. The included variables in this testing were as follows: “MoCA impaired performance,” “impaired motor performance” and “presence of depressive symptoms.” Starting from the weight matrix, after the integration of these new variables, the algorithm was repeated for each scale. The predictive ability of the modified scales was expressed in terms of sensitivity and specificity.

## Results

Results refer to 170 patients (mean age 77.7 ± 6.8 years, females *n* = 60, 35%) enrolled in Strat-AF study with complete clinical and brain MRI information. Table 1 shows the demographic and clinical characteristics at baseline.

**Table 1:** Demographic and clinical characteristics, cognitive, and motor performances; and depressive symptoms of the baseline Strat-AF study cohort (*n* = 170).

	**Score range**	**Total cohort**
Age (years)		77.7 ± 6.8
Gender (females)		60 (35%)
Schooling (years)		9.4 ± 4.3
Hypertension		140 (82%)
Diabetes		22 (13%)
Stroke		44 (26%)
Peripheral arterial pathology		14 (8%)
**Cognitive performance**		
MoCA (total score)	0–30	21.9 ± 3.9
MoCA impaired performance (ES = 0)		21 (12.5%)
**Motor performance**		
SPPB (total score)	0–12	9.5 ± 2.2
Impaired motor performance (SPPB score ≤ 10)		105 (62%)
**Depressive symptoms**		
GDS (total score)	0–15	3.44 ± 3.1
Presence of depressive symptoms (GDS score > 5)		38 (22%)

Table 2 shows the frequency distributions of CHA_2_DS_2_-VASc and HAS–BLED items. The mean total score was 3.7 ± 1.5 for CHA_2_DS_2_-VASc and 1.8 ± 0.8 for HAS–BLED. As per the inclusion criteria, all patients were older than 65 years, and 61% older than 75 years. Among the risk factors, 82% of patients had a history of hypertension, which was defined as uncontrolled in 10. Table 1 shows cognitive and motor performances and depressive symptoms at baseline.

**Table 2:** Frequency distributions of CHA_2_DS_2_-VASc and HAS–BLED items in the baseline Strat-AF study cohort (*n* = 170).

	**Score range**	**Total cohort**
CHA_2_DS_2_-VASc	0–9	3.7 ± 1.5
Age >75 years	0–2	61% (103/170)
Age 65**–**74 years	0–1	39% (67/170)
Sex category (female)	0–1	35% (60/170)
Congestive heart failure	0–1	15% (25/170)
Hypertension	0–1	82% (140/170)
Stroke and TIAs	0–2	26% (44/170)
Vascular disease	0–1	17% (29/170)
Diabetes	0–1	13% (22/170)
HAS–BLED	0–9	1.8 ± 0.8
Age >65 years	0–1	100% (170/170)
Uncontrolled hypertension	0–1	6% (10/170)
Abnormal renal function	0–1	6.5% (11/170)
Abnormal hepatic function	0–1	3% (5/170)
Stroke	0–1	22% (38/170)
Bleeding history or predisposition	0–1	10% (18/170)
Labile INR	0–1	6.5% (11/170)
Therapies	0–1	3% (5/170)
Alcohol	0–1	19% (32/170)

**Figure 1:**
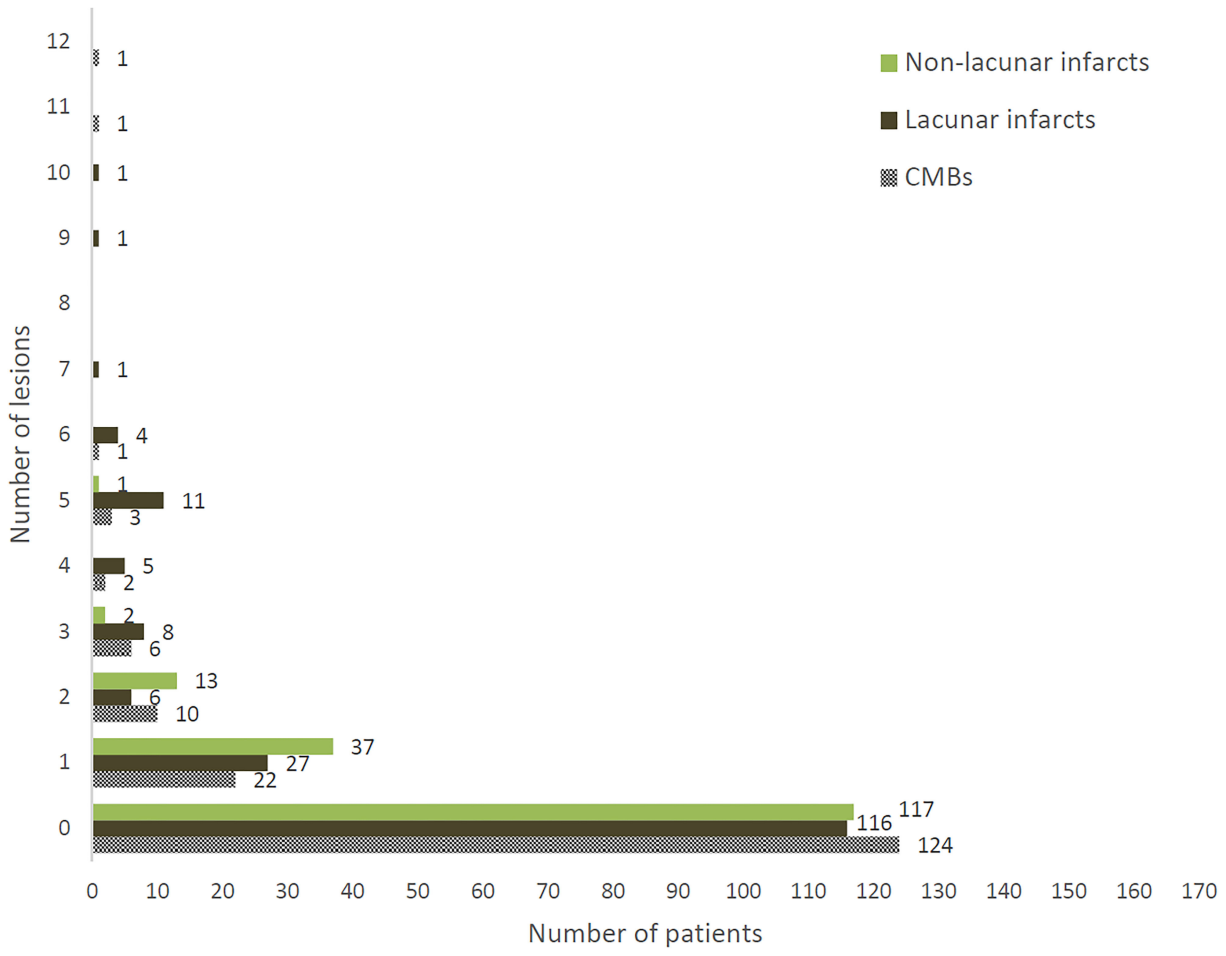
Distribution of number of CMBs, lacunar infarcts, and non-lacunar infarcts in 170 patients enrolled in Strat-AF study.

On brain MRI, 46 patients (27%) had at least one CMB, 54 (32%) at least one lacunar infarct, and 53 (31%) at least one non-lacunar infarct. Figure 1 shows distributions according to the lesion number for each outcome.

### Phase 1: Predictive Ability of CHA_2_DS_2_-VASc and HAS–BLED

The R algorithms returned sensitivity and specificity for all possible combinations of scores assumed by each scale. According to our data, the score ranges went from 0, i.e., absence of all clinical variables, to the maximum value that the scale would assume when all clinical variables were simultaneously present. As reported in Table 3, both scales had a poor predictive ability for each outcome as shown by sensitivity and specificity values.

**Table 3:** Sensitivity and specificity of CHA_2_DS_2_-VASc and HAS–BLED scales in relation to the three neuroimaging outcomes.

		**CMBs**		**Lacunar infarcts**		**Non-lacunar infarcts**	
	**Cutoff**	**Sens**	**Spec**	**Sens**	**Spec**	**Sens**	**Spec**
CHA_2_DS_2_–VASc	0	1	0	1	0	1	0
	1	0.9565	0.0323	1	0.0517	1	0.0513
	2	0.8696	0.2661	0.8889	0.2845	0.8679	0.2735
	3	0.6087	0.4919	0.6667	0.5259	0.7358	0.5556
	4	0.3043	0.7258	0.3518	0.75	0.566	0.8461
	5	0.087	0.8629	0.2037	0.9138	0.2641	0.9402
	6	0.0217	0.9274	0.0926	0.9569	0.1321	0.9744
	7	0	0.9919	0	0.9914	0.0189	1
	8	0	1	0	1	0	1
HAS–BLED	0	1	0	1	0	1	0
	1	0.4783	0.4516	0.6296	0.5172	0.6981	0.547
	2	0.1739	0.7903	0.2963	0.8448	0.3396	0.8632
	3	0.0217	0.9597	0.0556	0.9741	0.0377	0.9658
	4	0	1	0	1	0	1

### Phase 2: Changing Weights of CHA_2_DS_2_-VASc and HAS–BLED Items

Table 4 reports CHA_2_DS_2_-VASc and HAS–BLED accuracy indexes computed by our R algorithms, maximizing sensitivity (see “Methods” section) for each neuroimaging outcome. Overall, the predictive ability was not improved since the sensitivity values ranged from about 0.61–0.63 for CHA_2_DS_2_-VASc and from about 0.24–0.60 for HAS–BLED.

**Table 4:** Sensitivity and specificity of re-weighted CHA_2_DS_2_-VASc and HAS–BLED scales in relation to the three neuroimaging outcomes.

	**CMBs**		**Lacunar infarcts**		**Non-lacunar infarcts**	
	**CHA_**2**_DS_**2**_-VASc**	**HAS–BLED**	**CHA_**2**_DS_**2**_-VASc**	**HAS–BLED**	**CHA_**2**_DS_**2**_-VASc**	**HAS–BLED**
Sensitivity	0.6087	0.239	0.6296	0.5185	0.6226	0.6038
Specificity	0.686	0.8952	0.6724	0.8017	0.8889	0.9316

Supplementary Tables 1–6 report the most excluded items within the best combinations obtained for each outcome. Briefly, for CMBs, no variable was excluded from CHA_2_DS_2_-VASc, while abnormal renal function, INR, drugs and alcohol were the mostly excluded from HAS–BLED. For lacunar infarcts, sex and diabetes were the items more frequently excluded from CHA_2_DS_2_-VASc while uncontrolled hypertension, abnormal hepatic function, and drugs were excluded from HAS–BLED. Finally, for non-lacunar infarcts, items more frequently excluded were age, hypertension, vascular disease, and diabetes from CHA_2_DS_2_-VASc, and bleeding history or predisposition, drugs, alcohol and age ≥ 75 years from HAS–BLED.

### Phase 3: Adding New Items to CHA_2_DS_2_-VASc and HAS–BLED

Table 5 reports CHA_2_DS_2_-VASc and HAS–BLED accuracy indexes after re-weighting the original items and after the addition of the clinical ones, i.e., MoCA, SPPB, and GDS.

**Table 5:** Sensitivity and specificity of re-weighted CHA_2_DS_2_-VASc and HAS–BLED scales with the addition of MoCA, SPPB, and GDS in relation to the three neuroimaging outcomes.

	**CMBs**		**Lacunar Infarcts**		**Non-Lacunar Infarcts**	
	**CHA_**2**_DS_**2**_-VASc**	**HAS–BLED**	**CHA_**2**_DS_**2**_-VASc**	**HAS–BLED**	**CHA_**2**_DS_**2**_-VASc**	**HAS–BLED**
Sensitivity	0.6739	0.239	0.7407	0.5185	0.6981	0.6415
Specificity	0.6371	0.8952	0.6121	0.8103	0.8547	0.8974

Overall, HAS–BLED predictive ability was not improved since the sensitivity values still ranged from about 0.24 to 0.64. The CHA_2_DS_2_-VASc predictive ability for CMBs was confirmed as insufficient (sensitivity = 0.67, specificity = 0.64). However, CHA_2_DS_2_-VASc achieved an adequate sensitivity (0.74) but a low specificity (0.61) for lacunar infarcts and a sufficient predictive ability (sensitivity = 0.70, specificity = 0.85) for non-lacunar infarcts.

Supplementary Tables 8, 9 report the weights of the individual items for all the best combinations of re-weighted CHA_2_DS_2_-VASc with the addition of MoCA, SPPB, and GDS in relation to lacunar and non-lacunar infarcts. Focusing on the three new variables, MoCA was mostly excluded by the R algorithms while GDS retained a high weight for lacunar infarcts and SPPB for non-lacunar infarcts. As expected, the weights for stroke/TIA were consistently high across algorithms.

## Discussion

This study used *ad hoc* R algorithms to test the predictive ability of currently recommended risk stratification scales, CHA_2_DS_2_-VASc and HAS–BLED, on the presence of brain MRI lesions related to SVD and non-lacunar infarcts in elderly patients with AF on oral anticoagulants. Overall, the results showed a poor predictive ability of CHA_2_DS_2_-VASc and HAS–BLED for all outcomes. This was true when scoring the scales according to the original method (Phase 1) and also after changing the weights of each item (Phase 2). We also tested if the addition of the new clinical information, such as cognitive, motor and depressive scores, which are typical clinical hallmarks of cerebrovascular lesions, i.e., SVD markers and non-lacunar infarcts, could improve the performance indexes. Our results seem to point toward a potential role of motor and depressive scores in ameliorating CHA_2_DS_2_-VASc predictive ability for lacunar and non-lacunar infarcts. Un-expectedly, the global cognitive efficiency did not increase the predictive ability for any outcomes.

Data from our single center observational study are probably not surprising considering that CHA_2_DS_2_-VASc and HAS–BLED were not developed to predict imaging features of SVD. The prediction of such imaging features is a very complex issue, as is that of bleeding and thromboembolic events in elderly patients with AF who take anticoagulants. The topics should be addressed in large, multicentre, prospective studies, possibly with external validation in order to achieve possible answers to such difficult questions.

Concerning bleeding risk score, in Strat-AF we did not achieve any improvement for the hemorrhagic counterpart, i.e., HAS–BLED and CMB. Recently, the limitations of available bleeding risk scores have been the subject of discussion (27). Actually, no bleeding risk score has a clinical impact, i.e., physicians should not change the decision to prescribe anticoagulants in high stroke risk patients. The data of bleeding scores mainly derive from the patients on warfarin. Indeed, intracerebral bleeding risk has been reduced by the therapeutic use of direct oral anticoagulants. The possibility of improving the measurement of thrombotic risk is very important in order to better manage elderly patients avoiding no-treatment or under-treatment (low dosage oral anticoagulants or aspirin). From a clinical point of view, the improvement of the validity of thromboembolic risk stratification schemes on anticoagulant treatment decisions is of upmost relevance.

The research efforts are oriented toward the identification of the real predictive value of SVD-related MRI lesions, focusing on lesion load and possible threshold values, on the prediction of hemorrhagic and thromboembolic risk. The relevance of SVD-related MRI lesions on the prediction of an increased risk of intracerebral hemorrhage in patients with previous stroke and AF has been previously demonstrated in two studies, namely, CROMIS-2 and HERO (11, 12). Still, in asymptomatic elderly patients with AF available evidence does not support the routine use of brain MRI, also from a cost-effectiveness point of view. The development of a method able to identify the subgroup of patients that could maximally benefit from this “brain” additional information in risk stratification would be an optimal strategy for the real-world setting. The clinical variable we tested, cognitive, motor, and depressive scores, are based on widely used and easy to administer instruments.

The limitations of this study needs to be considered. First of all, the use of predictive algorithms required *ad hoc* computational resources able to estimate thousands of possible combinations, and such technical requirements limited our possibilities to add new clinical information to the risk stratification scales. Furthermore, our limited sample size and possible data overfitting impose caution in the interpretation of results. Neuroimaging outcomes could only be analysed as absent *vs*. present; thus, without considering the burden of multiple and/or coexistent lesions. Last but not the least, the data on motor performances and depressive symptoms have been registered in patients already on oral anticoagulants after AF diagnosis. Therefore, we do not know if they have a predictive role on MRI alterations. Further studies should confirm these results in AF populations anticoagulant–naive or with CHA_2_DS_2_-VASc scores 1 or 2 (males/females) for whom the net clinical benefit of anticoagulants is not clear.

Further investigations on large samples of patients are needed. The availability of big datasets would allow the use of artificial intelligence methods, e.g., machine and deep learning approaches. Moreover, instead of considering just clinical variables, the inclusion of circulating biological markers within these algorithms might augment the predictive ability. At present, we are carrying out a study that represents a continuation of the original Strat-AF. The Strat-AF 2 study (http://www.strat-af.it/2/) will offer the opportunity to analyze both longitudinal data, e.g., progression of cerebrovascular lesions burden, a wide pattern of circulating and omics biomarkers, and finally to test the new set of predictors.

Our results highlight the complexities about generating evidence to support precision medicine for elderly patients with AF (15). Brain MRI in the elderly patients with AF will not be considered in routine clinical practice until there are robust data to support the use of MRI biomarkers to predict the balance of risks of future ischemic and hemorrhagic events. In this sense, future research efforts are needed for the possible identification of elderly patients with AF who might benefit from the knowledge of additional information related to covert cerebrovascular burden.

## Data Availability Statement

The datasets presented in this article are not readily available because they are controlled by the Careggi University Hospital. Requests to access the datasets should be directed to AP, anna.poggesi@unifi.it.

## Ethics Statement

The studies involving human participants were reviewed and approved by Comitato Etico Area Vasta Centro, Regione Toscana. The patients/participants provided their written informed consent to participate in this study.

## Author Contributions

AP, ES, BB, EB, GDF, and RM: conceptualization. AP, ES, BB, EB, and GDF: methodology. EB, GDF, and BB: formal analysis. CB, BF, and SC: investigation. EB, GDF, CB, BF, SC, and ES: data curation. EB, GDF, AP, ES, and BB: original draft preparation. EF, FC, SD, BG, AG, SC, CM, FP, GP, CS, and RM: review and editing of manuscript. AP and BB: supervision. AP: funding acquisition. All authors have read and agreed to the published version of the manuscript.

## Funding

This research was funded by Tuscany region and Italian Ministry of Health under Grant Aimed Research Call “Bando Ricerca Finalizzata 2013” GR-2013-02355523; Title of the project, “Role of biological markers for cerebral risk stratification in patients with AF on oral anticoagulants for primary or secondary prevention of ischemic stroke”.

## Strat-af Study Group

### Coordinating Group

Anna Poggesi (Principal Investigator), Emilia Salvadori, Francesca Cesari, Rossella Marcucci, and Stefano Diciotti.

### Collaborators in Alphabetical Order

Carmen Barbato, Giorgio Busto, Eleonora Camilleri, Stefano Chiti, Samira Donnini, Enrico Fainardi, Benedetta Formelli, Francesco Galmozzi, Silvia Galora, Andrea Ginestroni, Betti Giusti, Anna Maria Gori, Elisa Grifoni, Invano Lombardo, Chiara Marzi, Anna Melone, Damiano Mistri, Marco Moretti, Francesca Pescini, Giovanni Pracucci, Valentina Rinnoci, Cristina Sarti.

## Conflict of Interest

The authors declare that the research was conducted in the absence of any commercial or financial relationships that could be construed as a potential conflict of interest.

## Publisher's Note

All claims expressed in this article are solely those of the authors and do not necessarily represent those of their affiliated organizations, or those of the publisher, the editors and the reviewers. Any product that may be evaluated in this article, or claim that may be made by its manufacturer, is not guaranteed or endorsed by the publisher.

## Supplementary Material

Supplementary Table 1 Weights of individual items, Youden index, cut-off, sensitivity, and specificity for all best combinations of re-weighted CHA_2_DS_2_-VASc in relation to CMBs (Phase 2).

Supplementary Table 2 Weights of individual items, Youden index, cut-off, sensitivity, and specificity for all best combinations of re-weighted CHA_2_DS_2_-VASc in relation to lacunar infarcts (Phase 2).

Supplementary Table 3 Weights of individual items, Youden index, cut-off, sensitivity, and specificity for all best combinations of re-weighted CHA_2_DS_2_-VASc in relation to non-lacunar infarcts (Phase 2).

Supplementary Table 4 Weights of individual items, Youden index, cut-off, sensitivity, and specificity for all best combinations of re-weighted HAS–BLED in relation to CMBs (Phase 2).

Supplementary Table 5 Weights of individual items, Youden index, cut-off, sensitivity, and specificity for all best combinations of re-weighted HAS–BLED in relation to lacunar infarcts (Phase 2).

Supplementary Table 6 Weights of individual items, Youden index, cut-off, sensitivity, and specificity for all best combinations of re-weighted HAS–BLED in relation to non-lacunar infarcts (Phase 2).

Supplementary Table 7 Weights of individual items, Youden index, cut-off, sensitivity, and specificity for all best combinations of re-weighted CHA_2_DS_2_-VASc with the addition of MoCA, SPPB, and GDS in relation to CMBs (Phase 3).

Supplementary Table 8 Weights of individual items, Youden index, cut-off, sensitivity, and specificity for all best combinations of re-weighted CHA_2_DS_2_-VASc with the addition of MoCA, SPPB, and GDS in relation to lacunar infarcts (Phase 3).

Supplementary Table 9 Weights of individual items, Youden index, cut-off, sensitivity, and specificity for all best combinations of re-weighted CHA_2_DS_2_-VASc with the addition of MoCA, SPPB, and GDS in relation to non-lacunar infarcts (Phase 3).

Supplementary Table 10 Weights of individual items, Youden index, cut-off, sensitivity, and specificity for all best combinations of re-weighted HAS–BLED with the addition of MoCA in relation to CMBs (Phase 3).

Supplementary Table 11 Weights of individual items, Youden index, cut-off, sensitivity, and specificity for all best combinations of re-weighted HAS–BLED with the addition of MoCA in relation to lacunar infarcts (Phase 3).

Supplementary Table 12 Weights of individual items, Youden index, cut-off, sensitivity, and specificity for all best combinations of re-weighted HAS–BLED with the addition of MoCA in relation to non-lacunar infarcts (Phase 3).
