# Supplementary material for: Can CHA2DS2-VASc and HAS–BLED Foresee the Presence of Cerebral Microbleeds, Lacunar and Non-Lacunar Infarcts in Elderly Patients With Atrial Fibrillation? Data From Strat–AF Study
Source: Front Neurol. 2022 May 13;13:883786. doi: 10.3389/fneur.2022.883786 (PMC9135961; doi:10.3389/fneur.2022.883786)
Supplement: Supplementary Table 1 — Weights of individual items, Youden index, cut-off, sensitivity, and specificity for all best combinations of re-weighted CHA2DS2-VASc in relation to CMBs (Phase 2). [file Data_Sheet_1.PDF]

Supplementary Materials

For the second and the third phases the algorithms returned a data structure for each outcome. Data structure is made by a number of rows equal to the number of selected weight combinations. The first part of the structure informs on weights that have to be assigned to the items to reach the maximum Youden index. The second part reports the associated cut-offs and related predictive ability indexes. When there is a large number of weight combinations, the output is limited only to the first 8 combinations.

Table S1: Weights of individual items, Youden index, cut-off, sensitivity and specificity for all best combinations of re-weighted CHA_2DS_2-VASc in relation to CMBs (Phase 2).

age	*sex*	*failure*	*hypertension*	*stroke*	*vascular*	*diabetes*	*Youden*	*cut-off*	*sens*	*spec*
1	2	4	1	1	3	4	0.2942	5	0.6087	0.686

Table S2: Weights of individual items, Youden index, cut-off, sensitivity and specificity for all best combinations of re-weighted CHA_2DS_2-VASc in relation to lacunar infarcts (Phase 2).

age	*sex*	*failure*	*hypertension*	*stroke*	*vascular*	*diabetes*	*Youden*	*cut-off*	*sens*	*spec*
1	0	2	1	2	1	0	0.302	3	0.6296	0.6724
1	0	2	1	3	1	0	0.302	3	0.6296	0.6724
1	0	2	1	4	1	0	0.302	3	0.6296	0.6724
1	0	3	1	2	2	0	0.302	4	0.6296	0.6724
1	0	3	1	3	2	0	0.302	4	0.6296	0.6724
1	0	3	1	4	2	0	0.302	4	0.6296	0.6724
1	0	3	2	2	1	0	0.302	4	0.6296	0.6724
1	0	3	2	3	1	0	0.302	4	0.6296	0.6724
…	…	…	…	…	…	…	0.302	…	0.6296	0.6724

Table S3: Weights of individual items, Youden index, cut-off, sensitivity and specificity for all best combinations of re-weighted CHA_2DS_2-VASc in relation to non-lacunar infarcts (Phase 2).

age	*sex*	*failure*	*hypertension*	*stroke*	*vascular*	*diabetes*	*Youden*	*cut-off*	*sens*	*spec*
0	1	1	0	1	0	0	0.5115	1	0.6226	0.8889
0	1	1	0	2	0	0	0.5115	1	0.6226	0.8889
0	1	1	0	3	0	0	0.5115	1	0.6226	0.8889
0	1	1	0	4	0	0	0.5115	1	0.6226	0.8889
0	1	1	1	2	0	0	0.5115	2	0.6226	0.8889
0	1	1	1	3	0	0	0.5115	2	0.6226	0.8889
0	1	1	1	4	0	0	0.5115	2	0.6226	0.8889
0	1	1	2	2	0	0	0.5115	3	0.6226	0.8889
…	…	…	…	…	…	…	0.5115	…	0.6226	0.8889

Table S4: Weights of individual items, Youden index, cut-off, sensitivity and specificity for all best combinations of re-weighted HAS-BLED in relation to CMBs (Phase 2).

hypertension	*renal*	*hepatic*	*stroke*	*bleeding*	*INR*	*therapies*	*alcohol*	*age75*	*Youden*	*cut-off*	*sens*	*spec*
4	0	2	2	3	0	0	0	1	0.1343	3	0.239	0.8952

Table S5: Weights of individual items, Youden index, cut-off, sensitivity and specificity for all best combinations of re-weighted HAS-BLED in relation to lacunar infarcts (Phase 2).

hypertension	*renal*	*hepatic*	*stroke*	*bleeding*	*INR*	*therapies*	*alcohol*	*age75*	*Youden*	*cut-off*	*sens*	*spec*
0	2	0	3	1	1	0	1	1	0.3202	2	0.5185	0.8017
0	2	0	4	1	1	0	1	1	0.3202	2	0.5185	0.8017
0	2	0	4	1	1	0	1	2	0.3202	3	0.5185	0.8017
0	2	1	3	1	1	0	1	1	0.3202	2	0.5185	0.8017
0	2	1	4	1	1	0	1	1	0.3202	2	0.5185	0.8017
0	2	1	4	1	1	0	1	2	0.3202	3	0.5185	0.8017
0	3	0	3	1	1	0	1	1	0.3202	2	0.5185	0.8017
0	3	0	4	1	1	0	1	1	0.3202	2	0.5185	0.8017
…	…	…	…	…	…	…	…	…	0.3202	…	0.5185	0.8017

Table S6*:* Weights of individual items, Youden index, cut-off, sensitivity and specificity for all best combinations of re-weighted HAS-BLED in relation to non-lacunar infarcts (Phase 2).

hypertension	*renal*	*hepatic*	*stroke*	*bleeding*	*INR*	*therapies*	*alcohol*	*age75*	*Youden*	*cut-off*	*sens*	*spec*
2	1	2	3	0	1	0	0	0	0.5354	2	0.6038	0.9316
2	1	2	3	0	1	1	0	0	0.5354	2	0.6038	0.9316
2	1	2	3	0	1	2	0	0	0.5354	2	0.6038	0.9316
2	1	2	3	1	1	0	0	0	0.5354	2	0.6038	0.9316
2	1	2	3	1	1	1	0	0	0.5354	2	0.6038	0.9316
2	1	2	4	0	0	0	1	1	0.5354	3	0.6038	0.9316
2	1	2	4	0	0	1	1	1	0.5354	3	0.6038	0.9316
2	1	2	4	0	1	0	0	0	0.5354	2	0.6038	0.9316
…	…	…	…	…	…	…	…	…	0.5354	…	0.6038	0.9316

Table S7: Weights of individual items, Youden index, cut-off, sensitivity and specificity for all best combinations of re-weighted CHA_2DS_2-VASc with the addition of MoCA, SPPB and GDS in relation to CMBs (Phase 3).

age	sex	failure	*hypertension*	stroke	vascular	diabetes	moca	sppb	gds	Youden	cut-off	sens	spec
1	1	4	2	1	3	4	1	1	0	0.311	6	0.6739	0.6371

Table S8: Weights of individual items, Youden index, cut-off, sensitivity and specificity for all best combinations of re-weighted CHA_2DS_2-VASc with the addition of MoCA, SPPB and GDS in relation to lacunar infarcts (Phase 3).

age	sex	failure	*hypertension*	stroke	vascular	diabetes	moca	sppb	gds	Youden	cut-off	sens	spec
2	0	4	2	2	1	1	0	1	3	0.3528	7	0.7407	0.6121
2	0	4	2	3	1	1	0	1	3	0.3528	7	0.7407	0.6121
2	0	4	2	4	1	1	0	1	3	0.3528	7	0.7407	0.6121

Table S9: Weights of individual items, Youden index, cut-off, sensitivity and specificity for all best combinations of re-weighted CHA_2DS_2-VASc with the addition of MoCA, SPPB and GDS in relation to non-lacunar infarcts (Phase 3).

age	sex	failure	*hypertension*	stroke	vascular	diabetes	moca	sppb	gds	Youden	cut-off	sens	spec
1	2	2	0	4	0	4	0	3	1	0.5528	8	0.6981	0.8547

For Phase 3 R algorithms, MoCA was the only clinical variable integrated within the HAS-BLED scale due to the burden of the computational process which to date is not supported by the available equipment.

Table S10: Weights of individual items, Youden index, cut-off, sensitivity and specificity for all best combinations of re-weighted HAS-BLED with the addition of MoCA in relation to CMBs (Phase 3).

hypert.	renal	hepatic	stroke	bleeding	*INR*	therapies	alcohol	age75	moca	Youden	cut-off	sens	spec
4	0	2	2	3	0	0	0	1	0	0.1343	3	0.239	0.8952

Table S11: Weights of individual items, Youden index, cut-off, sensitivity and specificity for all best combinations of re-weighted HAS-BLED with the addition of MoCA in relation to lacunar infarcts (Phase 3).

hypert.	renal	hepatic	stroke	bleeding	*INR*	therapies	alcohol	age75	moca	Youden	cut-off	sens	spec
1	4	0	3	1	0	0	1	1	1	0.3289	2	0.5185	0.8103
1	4	0	4	1	0	0	1	1	1	0.3289	2	0.5185	0.8103
1	4	0	4	1	0	0	1	2	1	0.3289	3	0.5185	0.8103
1	4	0	4	1	0	0	2	1	1	0.3289	3	0.5185	0.8103
1	4	0	4	2	0	0	2	1	1	0.3289	3	0.5185	0.8103
1	4	0	4	2	1	0	1	1	1	0.3289	3	0.5185	0.8103
1	4	0	4	2	1	0	2	0	1	0.3289	3	0.5185	0.8103
1	4	0	4	2	1	1	2	0	1	0.3289	3	0.5185	0.8103
…	…	…	…	…	…	…	…	…	…	…	…	…	…

Table S12: Weights of individual items, Youden index, cut-off, sensitivity and specificity for all best combinations of re-weighted HAS-BLED with the addition of MoCA in relation to non-lacunar infarcts (Phase 3).

hypert.	renal	hepatic	stroke	bleeding	*INR*	therapies	alcohol	age75	moca	Youden	cut-off	sens	spec
2	1	0	3	0	2	0	1	0	2	0.5389	2	0.6415	0.8974
2	1	0	3	0	2	1	1	0	2	0.5389	2	0.6415	0.8974
2	1	0	4	0	2	0	1	0	2	0.5389	2	0.6415	0.8974
2	1	0	4	0	2	1	1	0	2	0.5389	2	0.6415	0.8974
2	1	1	3	0	2	0	1	0	2	0.5389	2	0.6415	0.8974
2	1	1	3	0	2	1	1	0	2	0.5389	2	0.6415	0.8974
2	1	1	4	0	2	0	1	0	2	0.5389	2	0.6415	0.8974
2	1	1	4	0	2	1	1	0	2	0.5389	2	0.6415	0.8974
…	…	…	…	…	…	…	…	…	…	0.5389	…	0.6415	0.8974
